# Short repetition time diffusion-weighted imaging improves visualization of prostate cancer

**DOI:** 10.1007/s11604-023-01519-7

**Published:** 2023-12-21

**Authors:** Atsushi Higaki, Tsutomu Tamada, Ayumu Kido, Mitsuru Takeuchi, Kentaro Ono, Yoshiyuki Miyaji, Koji Yoshida, Hiroyasu Sanai, Kazunori Moriya, Akira Yamamoto

**Affiliations:** 1 Department of Radiology, Kawasaki Medical School, 577 Matsushima, Kurashiki City, Okayama Japan; 2 Department of Radiology, Radiolonet Tokai, Nagoya, 460-8501 Japan; 3 Department of Urology, Kawasaki Medical School, 577 Matsushima, Kurashiki City, Okayama Japan

**Keywords:** Apparent diffusion coefficient, Repetition time, Diffusion-weighted imaging, Magnetic resonance imaging, Prostate cancer

## Abstract

**Purpose:**

This study aimed to assess whether short repetition time (TR) diffusion-weighted imaging (DWI) could improve diffusion contrast in patients with prostate cancer (PCa) compared with long TR (conventional) reference standard DWI.

**Materials and methods:**

Our Institutional Review Board approved this retrospective study and waived the need for informed consent. Twenty-five patients with suspected PCa underwent multiparametric magnetic resonance imaging (mp-MRI) using a 3.0-T system. DWI was performed with TR of 1850 ms (short) and 6000 ms (long) with b-values of 0, 1000, and 2000s/mm^2^. Signal-to-noise ratio (SNR), contrast-to-noise ratio (CNR), visual score, apparent diffusion coefficient (ADC), and diagnostic performance were compared between short and long TR DWI for both b-values. The statistical tests included paired t-test for SNR and CNR; Wilcoxon signed-rank test for VA; Pearson's correlation and Bland–Altman plot analysis for ADC; and McNemar test and receiver operating characteristic analysis and Delong test for diagnostic performance.

**Results:**

Regarding b1000, CNR and visual score were significantly higher in short TR compared with long TR (*P* = .003 and *P* = .002, respectively), without significant difference in SNR (*P* = .21). Considering b2000, there was no significant difference in visual score between short and long TR (*P* = .07). However, SNR and CNR in long TR were higher (*P* = .01 and *P* = .04, respectively). ADC showed significant correlations, without apparent bias for ADC between short and long TR for both b-values. For diagnostic performance of DWI between short and long TR for both b-values, one out of five readers noted a significant difference, with the short TR for both b-values demonstrating superior performance.

**Conclusions:**

Our data showed that the short TR DWI_1000_ may provide better image quality than did the long TR DWI_1000_ and may improve visualization and diagnostic performance of PCa for readers.

## Introduction

The European Society of Urogenital Radiology (ESUR) prostate committee promoted the use of multi-parametric magnetic resonance imaging (mp-MRI) for routine magnetic resonance (MR) examination in patients with suspected or confirmed prostate cancer (PCa) in 2012 [1], which is widely accepted now. The main purpose of mp-MRI is to detect clinically significant PCa (csPCa). Detecting csPCa is crucial to reduce the number of biopsies and patients over-diagnosed with low-risk diseases and allow for appropriately directed biopsies for improving risk stratification. Radiologic-pathologic correlations with whole-mounts have shown that mp-MRI was highly sensitive for locating aggressive cancers, with 80–86% of Gleason score (GS) 7 and 93–100% of GS ≥ 8 detected [2]. However, it has been reported that mp-MRI missed less than 10% of csPCa on a per-lesion basis [2–6]. This implies that less than 10% of csPCa were missed if adopting only a targeted approach.

Mp-MRI is evaluated by a combination of diffusion-weighted imaging (DWI) and Apparent Diffusion Coefficient (ADC) maps, T2-weighted (T2WI), and dynamic contrast-enhanced (DCE) MRI. According to the Prostate Imaging Reporting and Data System (PI-RADS) v2.1, DWI and ADC maps are key components of mp-MRI in the prostate due to the high lesion contrast [7]. Therefore, DWI with a b-value of at least 1400 s/mm^2^ is recommended for sufficient lesion contrast. Furthermore, a low b-value of a minimum of 0–100 s/mm^2^ (preferably 50–100 s/mm^2^ and maximum of ≤ 1000 s/mm^2^ to avoid diffusion kurtosis effect are recommended for ADC calculation. Recently, it has been shown that T1 value may help differentiating PCa from normal prostate tissue (NPT) [8–13]. The T1 value is typically higher in cancer lesions than in normal tissue in other organs. However, in the prostate, there is a specific feature: the T1 value of PCa is lower than that of NPT. This specific characteristic suggests that by shortening the repetition time (TR), there might be potentials for enhanced T1 contrast in the prostate. Consequently, T1 shine through effect might also be expected to enhance DWI contrast. Therefore, in this study, we aimed to assess whether DWI with a shorter repetition time (TR) could improve diffusion contrast and diagnostic performance compared with DWI with long (conventional) TR according to our hospital’s routine protocol as a reference standard utilizing the specific feature of T1 in the prostate. Furthermore, we aimed to compare ADC calculated from DWI with short TR to ADC calculated from DWI with long (conventional) TR as reference standard. This new approach for improving diffusion contrast in the prostate may contribute to improving image quality and lesion detection.

## Materials and methods

### Participants

Our Institutional Review Board approved this retrospective study (approval number: 3850) and waived the need for informed consent. We initially identified the records of 41 consecutive patients with suspected PCa who underwent prostate mp-MRI at 3 T including DWI with short TR between December 2019 and June 2020. The exclusion criteria included cases of histologically unproven PCa on MRI-ultrasound (MRI-US) fusion-guided prostate-targeted biopsy (MRGB) for lesions suggestive of PCa on mp-MRI; post-treatment prostate; poor image quality; and incomplete MRI examination. Accordingly, 1 and 15 out of these 41 patients were excluded due to incomplete MRI examination and being—without MRGB-proven prostate lesions, respectively. Thus, 25 patients with PCa (n = 25) were included in the study. Additionally, nine of these patients underwent radical prostatectomy. No patient had undergone any therapy for PCa at the time of the MRI examination. The clinical features of the study population are summarized in Table 1.

**Table 1:** Summary of patient characteristics

Item	Value
Number of patients	25
Clinically significant prostate cancer lesions	25
Peripheral zone (PZ) lesions	15
Transition zone (TZ) lesions	10
Age (years)	
Mean ± SD	70.6 ± 7.34
Range	54–86
Initial PSA level (ng/mL)	
Mean ± SD	37.0 ± 105
Range	1.78–533
Interval between MRI examination and MRGB (days)	
Mean ± SD	38.3 ± 33.0
Range	2–122
Gleason score	
3 + 3	5
3 + 4	7
4 + 3	7
4 + 4	2
4 + 5	4

*MRGB* MRI-ultrasonography fusion-guided prostate-targeted biopsy; *PSA* prostate-specific antigen; *SD* standard deviation

### MRI

All MR images were obtained with a 3.0-T MRI (Ingenia 3.0 T CX; Philips Healthcare, Best, The Netherlands) using an anterior and built-in posterior coils. In all patients, peristalsis was suppressed with an intramuscular injection of 20 mg of scopolamine butylbromide (Buscopan, Boehringer Ingelheim Pharmacy, Yamagata, Japan) or 1 mg of glucagon (Glucagon-G Novo, Eisai Pharmacy, Tokyo, Japan) before beginning MR examination. Examinations included DWI, T1 mapping, T2-weighted imaging (T2WI), T1-weighted imaging (T1WI), 3D T2WI, and DCE MRI. DWI, T2WI, and T1 mapping were used for assessing PCa lesions, identifying the anatomical location of lesions, and measurement of T1 values, respectively. T1-weighted imaging (T1WI), 3D T2WI, and DCE MRI were not assessed as they were unnecessary for this study.

DWI was performed using spin-echo type single-shot echo planar imaging with TR of 1850 ms (short) and 6000 ms (long). DWI with TR of 6000 ms is used as a routine protocol for prostate DWI in our hospital and represents the reference standard in this article. The detailed imaging parameters for short and long TR DWI are described in Table 2.

**Table 2:** Acquisition parameters for DWI with short and long TR

Parameters	Short TR DWI	Long TR DWI
Field of view (FOV) (mm)	300 × 300	300 × 300
Number of slices	30	30
TR (ms)	1850	6000
TE (ms)	70	70
Acquired voxel size (mm^3^)	3.13 × 3.13 × 3.00	3.13 × 3.13 × 3.00
SENSE reduction factor	2	2
Number of packages	3	1
b-value (s/mm^2^)	0, 1000, 2000	0, 1000, 2000
Number of signals averaged (NSA)	2 (b0), 4 (b1000), 8 (b2000)	2 (b0), 4 (b1000), 8 (b2000)
Scan time	3 min 50 s	3 min 50 s

Number of packages: the number of acquisitions classified in 2D multi-slice imaging

*DWI* diffusion-weighted imaging; *TE* echo time; *TR* repetition time

Transverse turbo spin echo (TSE) T2WI was acquired with the following parameters: TR, 7257 ms; echo time (TE), 95 ms; a field of view (FOV), 200 mm; acquisition voxel size, 0.57 × 0.72 × 3.0 mm^3^; TSE factor, 9; number of signals averaged (NSA), 1; and SENSE reduction factor, 1.4. The scan duration was 5 min and 27 s.

T1 map was calculated using 3D Look-Locker acquisition [14] with the following parameters: TR / TE, 1.97 ms / 0.92 ms; flip angle (FA), 7°; phase interval, 95 ms; phase number, 20; recovery period, 5000 ms; slices, 8; and scan time, 56 s.

### MRGB

The UroStation system (Koelis; Grenoble, France) with elastic image fusion, real-time 3D organ-tracking technology, and a computer workstation (Koelis) for segmentation of the prostate and lesion under the local perianal muscle and transrectal ultrasound (TRUS)-guided periprostatic plexus anesthesia were used for performing all MRGB procedures [15]. A radiologist performed segmentation of the whole prostate and MRI-defined lesions based on 3D mp-MRI data (mostly T2WI) using the UroStation workstation before biopsy. The mp-MRI 3D volume data and real-time TRUS image were elastically fused on the screen. Immediately after biopsy, core of the target lesion displayed on the fusion image was obtained by biopsy needle under TRUS guidance. Additional real-time 3D TRUS images were obtained to determine the accuracy of needle deployment within the target lesion. MRI-target lesions were defined as assessment category 3 or higher lesion in PI-RADS v2 or PI-RADS v2.1 [7, 16]. At least two cores were obtained for each MRI-targeted lesion. Additional cores were obtained at the operator’s discretion based on lesion size and location and confidence in targeting accuracy. In addition to MRGB, we also performed systematic biopsies covering 10–12 locations.

### Histopathologic examination

The MRGB specimens were stained with hematoxylin and eosin. The radical prostatectomy specimens underwent standard step sectioning at 4–6-mm intervals, with subsequent hematoxylin and eosin staining. A pathologist recorded the presence or absence of PCa and tumor GS in biopsy specimens. Additionally, the tumor location, GS of all tumor foci, and extraprostatic extension locations on a standardized prostate diagram for prostatectomy specimens were recorded. GS was diagnosed according to the 2014 International Society of Urological Pathology Modified Gleason Grading System [17].

### Clinically significant prostate cancer

csPCa was defined as a tumor with GS ≥ 7 and tumor diameter ≥ 5 mm or with GS = 3 + 3 and tumor size ≥ 0.5 mL (tumor diameter ≥ 8 mm) [7]. Tumor size was calculated based on mp-MRI, most commonly T2WI.

### Data evaluation

#### Quantitative and qualitative analysis

The signal-to-noise ratio (SNR), contrast-to-noise ratio (CNR), and visual score were compared between DWI at a b-value of 1000 s/mm^2^ (DWI_1000_) with short TR and long TR and between DWI at a b-value of 2000s/mm^2^ (DWI_2000_) with short TR and long TR. In addition, the ADCs calculated by DWI with short TR at b-values of 0 and 1000 s/mm^2^ and b-values of 0 and 2000s/mm^2^ were compared with the DWI using long TR. Moreover, T1 values in PCa and NPT were compared to validate if there was a difference in T1 [8–13]. These quantitative analyses were performed by two radiologists (two fellowship-trained radiologists with 8 years (A.K.) and 12 years (A.Y.)) of experience in prostate MRI in consensus.

SNR in NPT was measured on short and long TR DWI for both b-values of 1000 and 2000s/mm^2^. A region of interest (ROI) was carefully selected in the normal peripheral zone (PZ) and left or right internal obturator muscle on the b0 image in reference to histopathological results and T2WI. Then, the ROIs were copied to the b1000 and b2000 images. The mean ROI size in NPT was 107 (range, 59–204) mm^2^. The mean ROI size in muscle was 205 (range, 197–215) mm^2^.

SNR was defined as follows:

$${\text{SNR}} = {\text{SI}}_{{\text{NPT}}} /{\text{SD}}_{{\text{muscle}}} .$$

SI_NPT_ and SD_muscle_ were the average signal intensity in NPT and standard deviation of the signal in muscle inside ROI, respectively.

CNR between PCa and NPT was measured in short and long TR DWI for both b-values. ROI in PCa was carefully selected in the lesion on the ADC map with b-values of 0 and 2000s/mm^2^ in reference to histopathological results and T2WI. The same ROI as SNR measurement was used for NPT and muscle. The mean ROI size in PCa was 85 (range, 8–571) mm^2^.

CNR was defined as:

$${\text{CNR}} = \left( {{\text{SI}}_{{\text{PCa}}} - {\text{SI}}_{{\text{NPT}}} } \right)/{\text{SD}}_{{\text{muscle}}} .$$

where SI_PCa_ and SI_NPT_ were the average signal intensity in PCa and NPT, and SD_muscle_ was the standard deviation of the signal in muscle inside the ROI.

This quantitative method is based on references from previous literature [18].

The visual score for comparison of contrast between PCa and NPT in short and long TR DWI for both b-values was assessed by a fellowship-trained radiologist with 22 years of experience in prostate MRI (T.T.). It was graded using a 3-point scale, where, 1 = weak; 2 = moderate; 3 = strong contrast.

ADCs at b-values of 0 and 1000 s/mm^2^ and b-values of 0 and 2000s/mm^2^ in short and long TR DWI were measured in PCa, NPT, and muscle. The same ROI as the CNR measurement was used for each tissue.

T1 value in PCa and NPT was measured. The same ROI as the CNR measurement was used for each tissue. Moreover, the T1 saturation recovery graph was drawn using the mean T1 value of twenty-five patients measured in PCa and NPT.

#### Diagnostic performance

Regarding diagnostic performance, we used the radical prostatectomy specimens as the reference standard. The diagnostic performance of DWI_1000_ with short and long TR and DWI_2000_ with short and long TR was compared in nine patients with histopathologically proven csPCa who underwent radical prostatectomy.

Three months after the visual score evaluation, four fellowship-trained radiologists with experience of 2 years (reader 1, K.O.), 8 years (reader 2, A.K.), 12 years (reader 3, A.Y.), and 22 years (reader 4, T.T.) in prostate MRI, along with a fellowship-trained urologist with 13 years of experience (reader 5, Y.M.) in prostate MRI, independently evaluated four different DW images using the PI-RADS v2.1 DWI score. Each reader interpreted the images separately for absolute evaluation. The prostate was classified into eight regions on transverse T2WI; six PZ as the right base, right mid-gland, right apex, left base, left mid-gland, and left apex and two transition zones (TZ) as right and left regions. The study coordinator (A.H.) prepared a PowerPoint file (Microsoft, Washington, USA) showing the slice number corresponds to the eight regions on the DWI for each patient prior to assessing DW images by the radiologists. Image evaluation was performed using only DWI and ADC maps blinded to T2-weighted imaging and DCE-MRI with reference to the PowerPoint file at a dedicated workstation (Synapse EX; Fujifilm, Tokyo, Japan). The readers were aware of the patient’s age but were blinded to prostate-specific antigen (PSA) level and histopathology results. Each radiologist independently assessed four different DW images with ADC maps and assigned scores of 1–5 for DWI for each region. If there are multiple lesions in each region, the lesion with the largest and strongest signal change was evaluated. The score was given for all areas of lesion covered multiple areas.

### Statistical analysis

A paired t-test for SNR and CNR and Wilcoxon signed-rank test for visual score between DWI_1000_ with short and long TR and DWI_2000_ with short and long TR were used for the statistical analysis. Pearson’s correlation was used for analyzing the correlation between ADCs at b-values of 0 and 1000 s/mm^2^ using short and long TR and between ADCs at b-values of 0 and 2000s/mm^2^ using short and long TR. The bias of ADCs at b-values of 0 and 1000 s/mm^2^ between short and long TR and ADCs at b-values of 0 and 2000s/mm^2^ between short and long TR were evaluated using Bland–Altman plot analysis. Paired t-test for the T1 value between PCa and NPT was used.

For csPCa detection, diagnostic sensitivity, specificity, positive predictive value, negative predictive value, and accuracy when positive for DWI score ≥ 3 in each region were measured for each DWI by each reader. The McNemar test was used for comparing sensitivity, specificity, and accuracy between DWI_1000_ with short and long TR and DWI_2000_ with short and long TR. Receiver operating characteristic (ROC) analysis was used for evaluating the diagnostic performance for csPCa detection on DWI_1000_ with short and long TR and DWI_2000_ with short and long TR. A comparison of the area under the curve (AUC) obtained from the ROC analysis was performed between DWI_1000_ with short and long TR and DWI_2000_ with short and long TR by each radiologist using the Delong test. The data by the four readers were then combined to analyze the number of lesions rated as PI-RADS DWI score 3 by conventional DW imaging using long TR and the degree to which these lesions were upgraded or downgraded by short TR.

All statistical analyses were performed using SPSS for Windows v. 24.0 software (SPSS, Chicago, IL) and JMP v. 11.0.0 software (SAS, Cary, NC). A *P* < .05 was considered significant.

## Results

### Quantitative and qualitative analysis

**Fig. 1:**
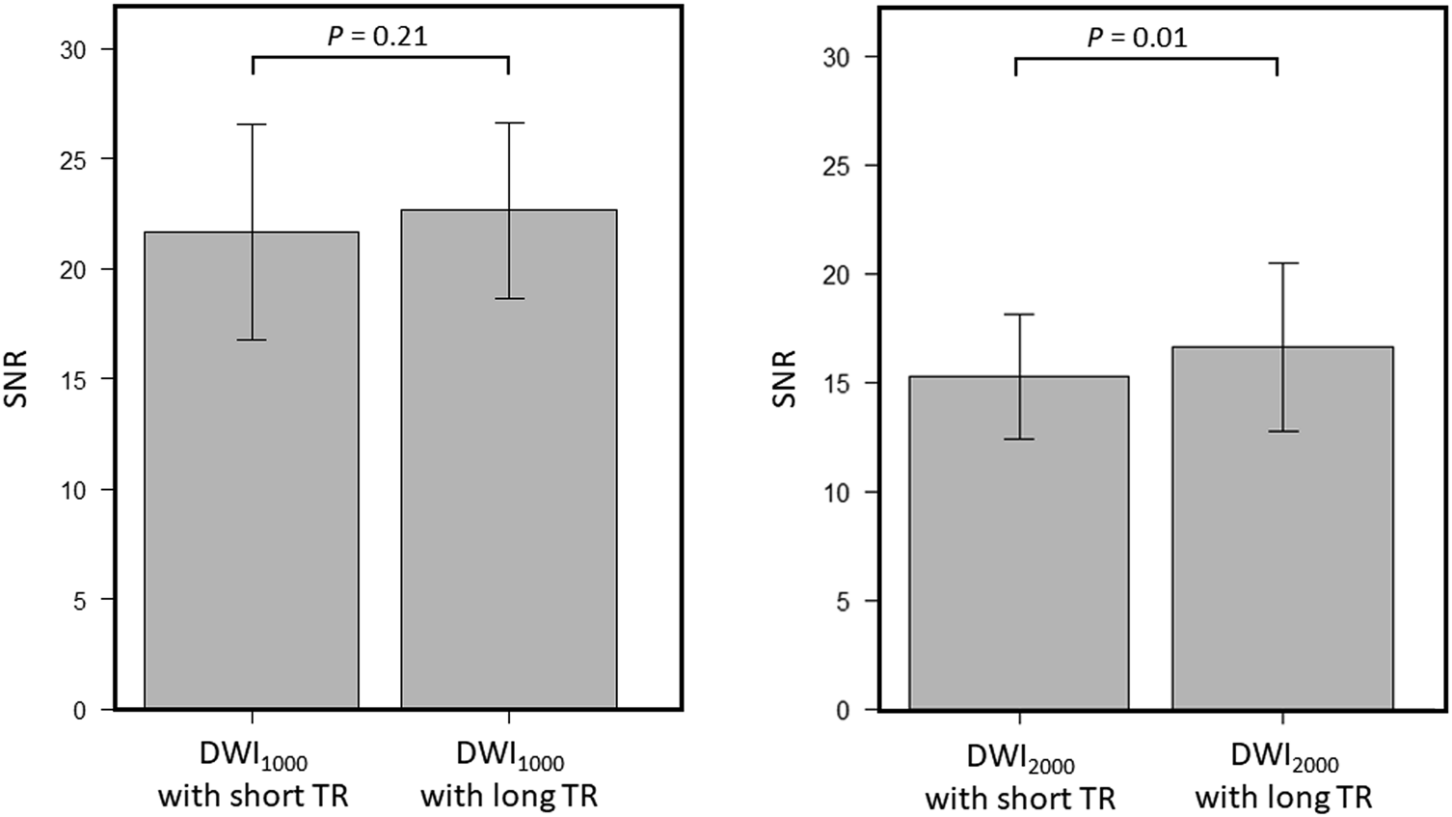
Comparison of the mean SNR between DWI at a b-value of 1000 s/mm^2^ (DWI_1000_) with short and long TR and between DWI at a b-value of 2000s/mm^2^ (DWI_2000_) with short and long TR. The mean SNR is 21.7 ± 4.70 (standard deviation [SD]) in short TR and 22.6 ± 3.94 in long TR for b1000, and 15.1 ± 2.77 in short TR and 16.6 ± 3.78 in long TR for b2000. The difference in SNR between DWI_1000_ with short and long TR is not significant (*P* = .21), whereas the difference between DWI_2000_ with short and long TR is significant (*P* = .01). The error bars indicate the SD in the 25 patients. DWI; diffusion-weighted imaging, TR; repetition time

**Fig. 2:**
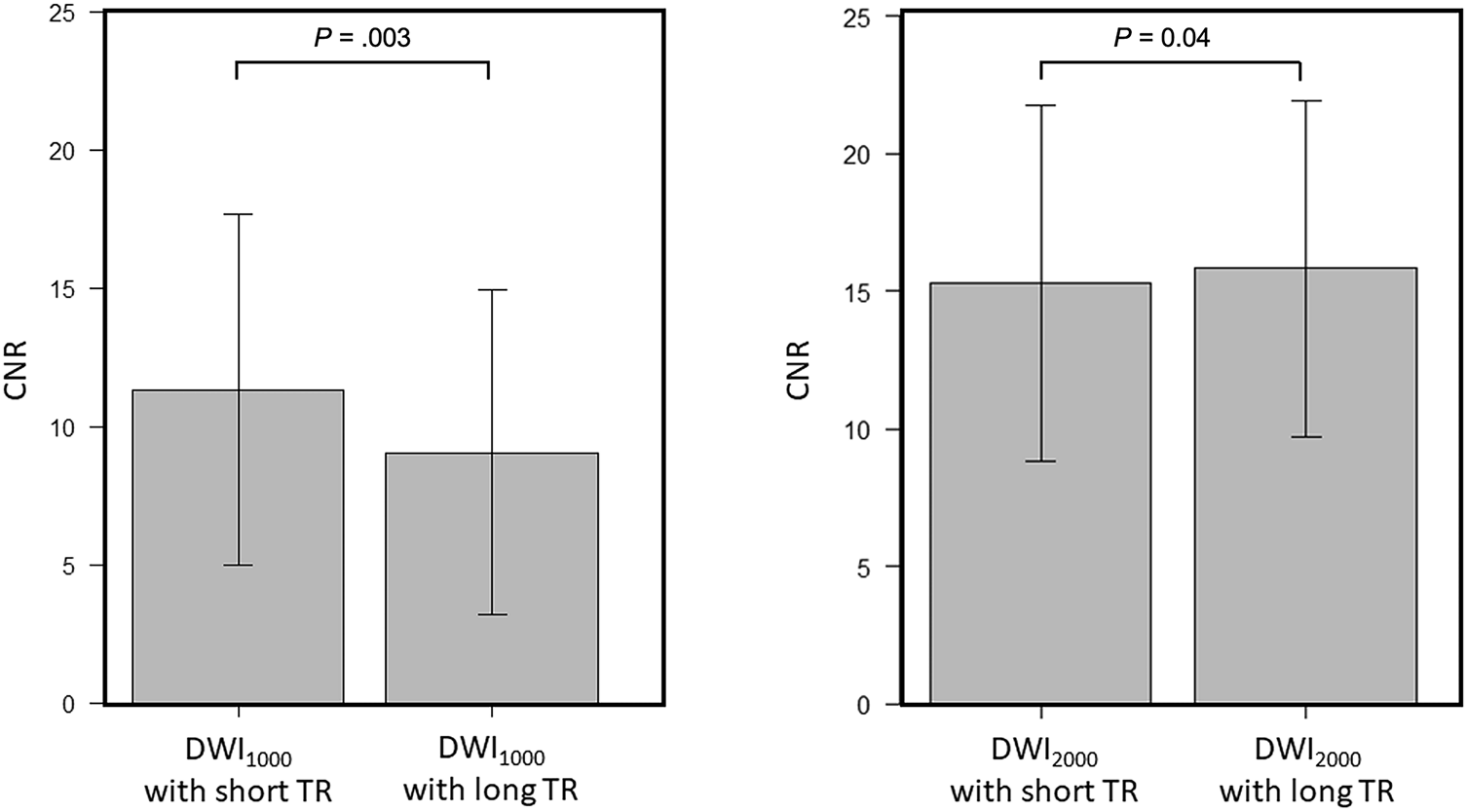
Comparison of the mean CNR between DWI at a b-value of 1000 s/mm^2^ (DWI_1000_) with short and long TR and between DWI at a b-value of 2000s/mm^2^ (DWI_2000_) with short and long TR. The mean CNR is 11.0 ± 6.27 (standard deviation [SD]) in short TR and 9.05 ± 5.75 in long TR for b1000, and 14.9 ± 6.52 in short TR and 15.8 ± 6.00 in long TR for b2000. The difference in CNR between DWI_1000_ with short and long TR and DWI_2000_ with short and long TR is significant (*P* = .003 and *P* = .04, respectively). The error bars indicate the SD in the 25 patients. CNR: contrast-to-noise ratio, DWI: diffusion-weighted imaging, TR: repetition time

**Fig. 3:**
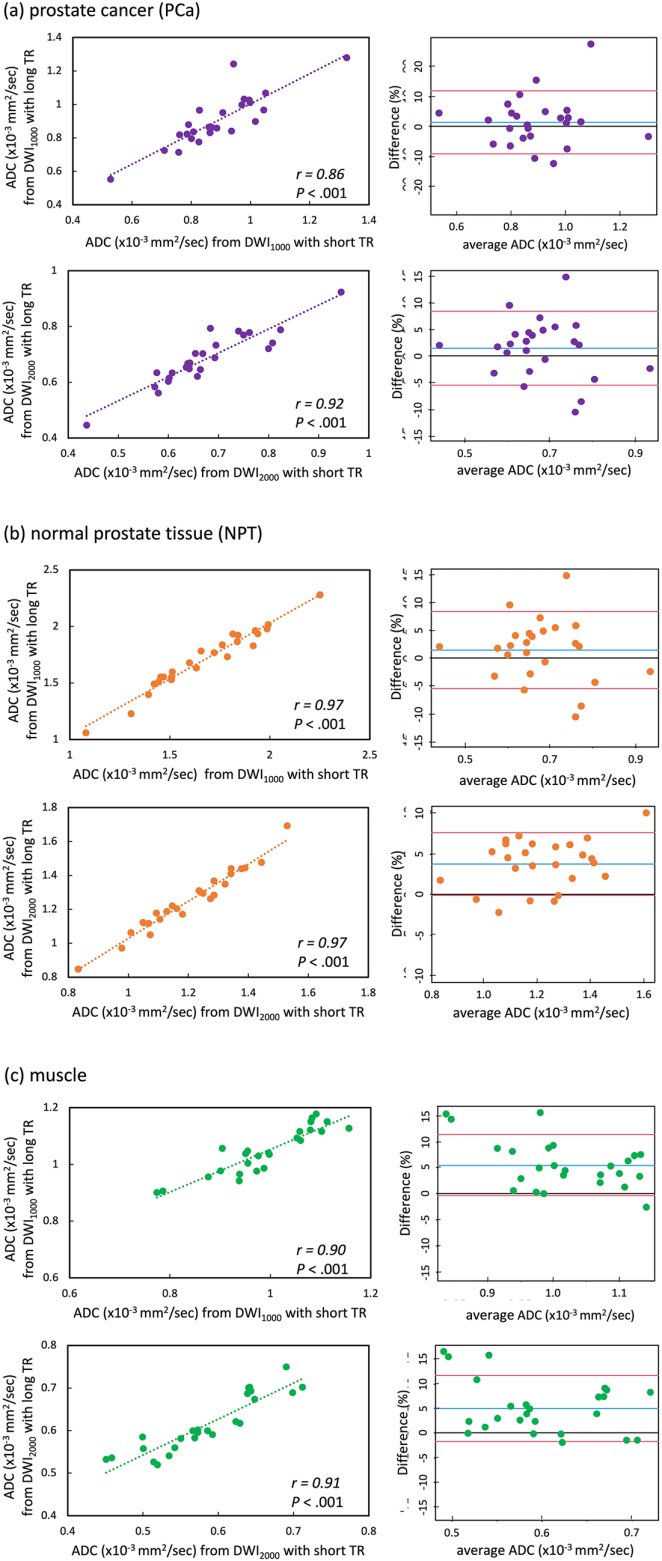
Scatter plots and Bland–Altman plots of ADCs in prostate cancer (PCa) (**a**), normal prostate tissue (NPT) (**b**), and muscle (**c**) between DWI at a b-value of 1000 s/mm^2^ (DWI_1000_) with short and long TR (upper row), and between DWI at a b-value of 2000s/mm^2^ (DWI_2000_) with short and long TR (lower row). Regarding both b-value, ADCs in PCa, NPT, and muscle show significantly strong correlations between short and long TR. The Bland–Altman plot analysis shows no apparent bias for ADCs in PCa, NPT, and muscle between short and long TR. In the Bland–Altman plot, the x-axis shows the average of two ADCs calculated by short and long TR DWI, and the y-axis shows the difference (%) between the two ADCs. The blue and red lines represent the mean bias and the limits of agreement (mean ± 1.96 × SD), respectively. ADC: apparent diffusion coefficient, DWI: diffusion-weighted imaging, TR: repetition time

**Fig. 4:**
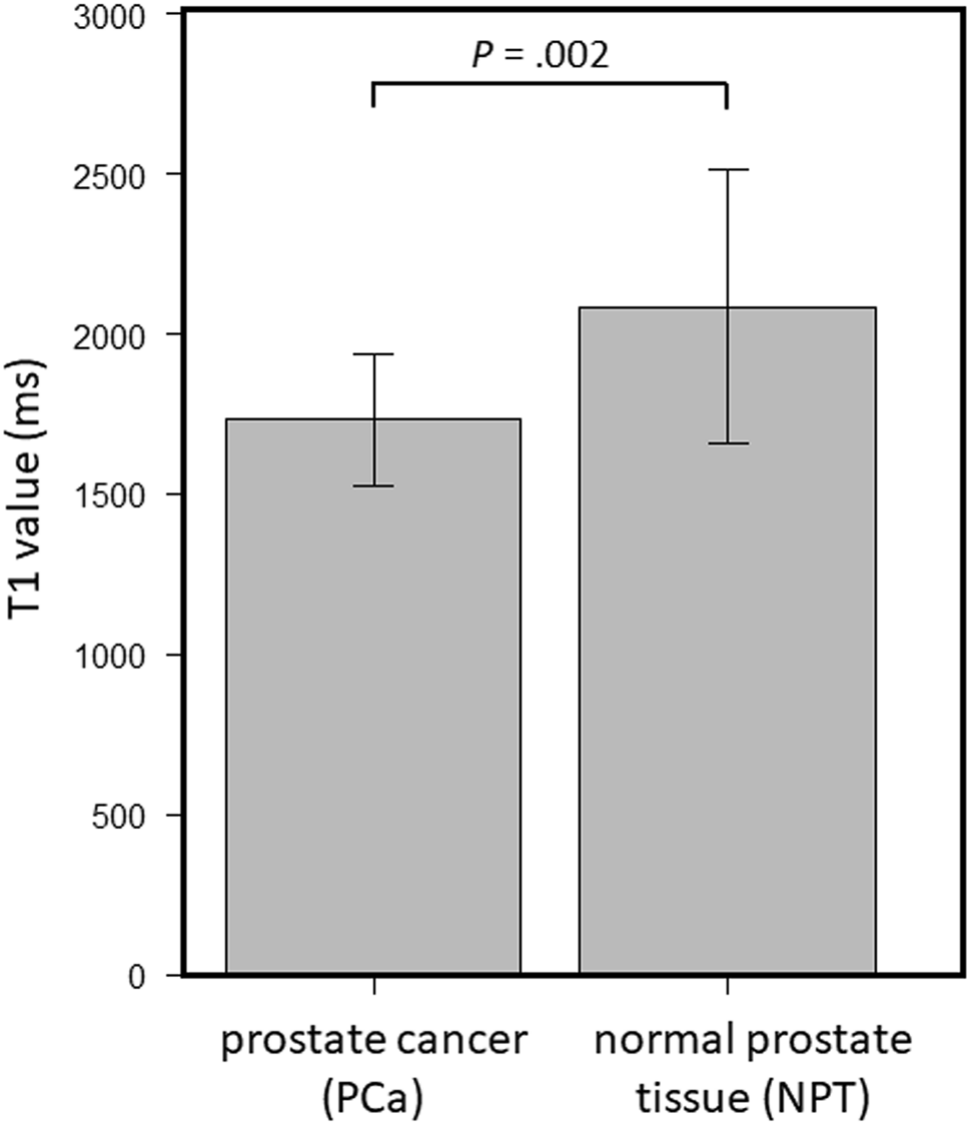
Comparison of the mean T1 value between prostate cancer (PCa) and normal prostate tissue (NPT). The mean T1 value was 1726 ± 194 (standard deviation [SD]) ms in PCa and 2086 ± 416 ms in NPT. The difference in T1 value between PCa and NPT is significant (*P* = .002). The error bars indicate the SD in the 25 patients

**Fig. 5:**
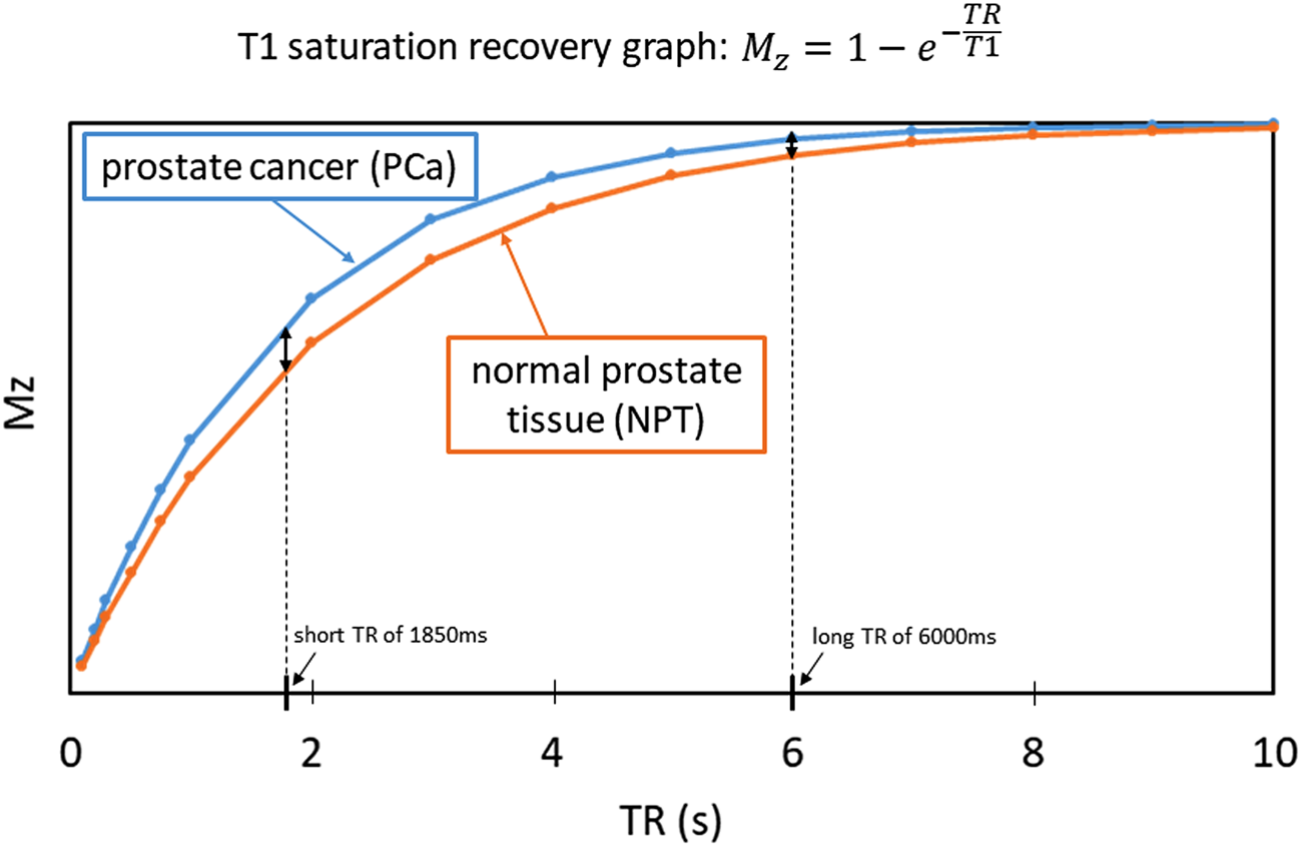
T1 saturation recovery graph. This graph is drawn using the T1 value of 1700 ms in PCa (blue line) and 2100 ms in NPT (orange line)

**Fig. 6:**
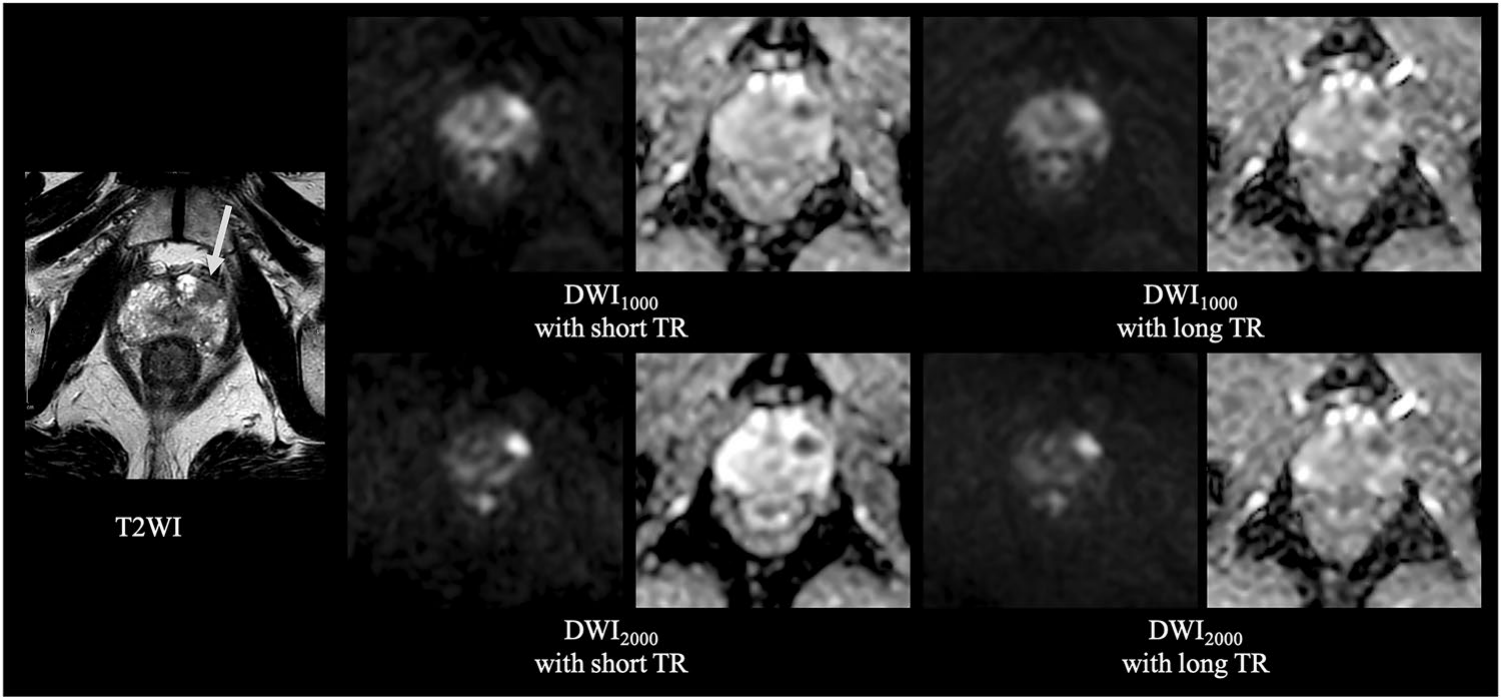
Representative images for T2WI, DWI at a b-value of 1000 s/mm^2^ (DWI_1000_) with short and long TR (upper row), and DWI at a b-value of 2000s/mm^2^ (DWI_2000_) with short and long TR (lower row). Images in a 63-year-old man with Gleason score 4 + 5 PCa (T2c; prostate-specific antigen [PSA]: 4.8 ng/mL). Transverse T2WI shows a low signal intensity lesion in the left peripheral zone (white arrow). DWI_1000_ with short TR shows better lesion contrast than DWI_1000_ with long TR, whereas DWI_2000_ with short TR shows comparable image quality compared to DWI_2000_ with long TR. DWI; diffusion-weighted imaging, TR; repetition time

A comparison between short and long TR acquisition with a b-value of 1000 s/mm^2^ revealed that there was no significant difference in the mean SNR (21.7 ± 4.70 standard deviation [SD] for short TR, 22.6 ± 3.94 for long TR, *P* = 0.21) (Fig. 1). However, the mean CNR and median visual score were significantly higher in short TR than in long TR (CNR; 11.0 ± 6.27 for short TR, 9.05 ± 5.75 for long TR, *P* = 0.003, visual score; 2 [2, 3] [25th percentile, 75th percentile] for short TR, 2 [1, 2] for long TR, *P* = 0.002) (Fig. 2 and Table 3). ADCs in PCa, NPT, and muscle showed significant strong correlations between short and long TR for b1000 (r = 0.86 and *P* < 0.001; r = 0.97 and *P* < 0.001; r = 0.90 and *P* < 0.001, respectively) (Fig. 3). The Bland–Altman plot analysis showed no noticeable bias for ADCs in PCa, NPT, and muscle between short and long TR for b1000 [bias = 1.45% and limits of agreement (mean ± 1.96 × SD) = − 8.92% to 11.8%; bias = 1.91% and limits of agreement = − 2.49–6.31%; bias = 5.54% and limits of agreement = − 0.26–11.3%] (Fig. 3). A comparison between short and long TR acquisition with a b-value of 2000s/mm^2^ revealed that there was no significant difference in the median visual score (3 [2, 3], 3 [2, 3], *P* = .07) (Table 3). However, long TR had higher mean SNR and CNR than did short TR (SNR; 15.1 ± 2.77 for short TR, 16.6 ± 3.78 for long TR, *P* = .01, CNR; 14.9 ± 6.52 for short TR, 15.8 ± 6.00 for long TR, *P* = .04) (Figs. 1 and 2). ADCs in PCa, NPT, and muscle showed significant strong correlations between short and long TR for b2000 (r = 0.92 and *P* < .001; r = 0.97 and *P* < .001; r = 0.91 and *P* < .001, respectively) (Fig. 3). The Bland–Altman plot analysis showed no apparent bias for ADCs in PCa, NPT, and muscle between short and long TR for b2000 (bias = 1.42% and limits of agreement = − 5.49–8.32%; bias = 3.79% and limits of agreement = − 0.03–7.61%; bias = 5.10% and limits of agreement = − 1.63–11.8%) (Fig. 3). The mean T1 value in PCa was significantly lower than that in NPT (1726 ± 194 for PCa, 2086 ± 416 for NPT, *P* = .002) (Fig. 4). The T1 saturation recovery graph calculated by T1 values of 1700 ms in PCa and 2100 ms in NPT is shown in Fig. 5. Representative images are shown in Fig. 6.

**Table 3:** Comparison of the median visual score between DWI at a b-value of 1000 s/mm^2^ (DWI_1000_) with short and long TR, and between DWI at a b-value of 2000s/mm^2^ (DWI_2000_) with short and long TR

	With short TR	With long TR	*P* value
DWI_1000_	2 [2, 3]	2 [2, 3]	.002
DWI_2000_	3 [2, 3]	3 [2, 3]	.07

The number indicates the median value [25th, 75th percentiles]

*DWI* diffusion-weighted imaging; *TR* repetition time

### Diagnostic performance

In a comparative analysis of the diagnostic performance of DWI_1000_ and DWI_2000_ under short and long TRs for detecting clinically significant prostate cancer, one out of the five readers (A urologist not specialized in prostate MRI reading) observed that short TR DWI consistently showcased a superior sensitivity, accuracy, and AUC throughout the entire prostate, covering both PZ and TZ, in contrast to long TR DWI. This notable difference was significant for both b-values (Table 4, *P* values ranging from < 0.001 to 0.041). In evaluations specific to the PZ, DWI_1000_ with a short TR displayed enhanced sensitivity over its long TR counterpart. Additionally, short TR DWI surpassed long TR DWI across both b-values in AUC metrics (Table 5, *P* values between 0.001 and 0.041). However, for assessments confined to the TZ, no discernible differences were found in terms of sensitivity, specificity, accuracy, or AUC (Table 6). In contrast, the evaluations from the remaining four readers revealed no significant discrepancies in AUC, sensitivity, specificity, or accuracy between DWI_1000_ (both short and long TR) and DWI_2000_ (both short and long TR). Comparison of visual rating scores via short TR DWI for PI-RADS DWI score 3 lesions diagnosed using conventional DWI with long TR is shown in Table 7. Using conventional DW imaging with long TR, 43 out of 360 cases (11.9%) were rated as PI-RADS DWI score 3 on DWI_1000_, while 26 out of 360 cases (7.2%) were rated as such on DWI_2000_. Moreover, 21 of 43 cases (48.8%) were upgraded or downgraded on DWI_1000_, and 11 of 26 cases (42.3%) on DWI_2000_, resulting in an improvement in accuracy.

**Table 4:** Comparison of diagnostic performance for clinically significant prostate cancer detection across the entire prostate, encompassing both PZ and TZ, between DWI_1000_ and DWI_2000_ with short and long TR

Region	Parameter	Reader	DWI_1000_ with short TR	DWI_1000_ with long TR	*P* value	DWI_2000_ with short TR	DWI_2000_ with long TR	*P* value
PZ + TZ	Sensitivity, %	1	88.5 (23/26)	88.5 (23/26)	n.s	88.5 (23/26)	88.5 (23/26)	n.s
		2	88.5 (23/26)	88.5 (23/26)	n.s	88.5 (23/26)	88.5 (23/26)	n.s
		3	80.8 (21/26)	80.8 (21/26)	n.s	80.8 (21/26)	84.6 (22/26)	n.s
		4	84.6 (22/26)	80.8 (21/26)	n.s	84.6 (22/26)	88.5 (23/26)	n.s
		5	84.6 (22/26)	53.8 (14/26)	0.01	92.3 (24/26)	69.2 (18/26)	0.04
	Specificity, %	1	91.3 (42/46)	89.1 (41/46)	n.s	89.1 (41/46)	91.3 (42/46)	n.s
		2	93.5 (43/46)	91.3 (42/46)	n.s	91.3 (42/46)	91.3 (42/46)	n.s
		3	93.5 (43/46)	93.5 (43/46)	n.s	93.5 (43/46)	93.5 (43/46)	n.s
		4	91.3 (42/46)	95.7 (44/46)	n.s	93.5 (43/46)	93.5 (43/46)	n.s
		5	89.1 (41/46)	80.4 (37/46)	n.s	84.8 (39/46)	71.7 (33/46)	n.s
	PPV, %	1	85.2 (23/27)	82.4 (23/28)	NA	82.4 (23/28)	85.2 (23/27)	NA
		2	88.5 (23/26)	85.2 (23/27)	NA	85.2 (23/27)	85.2 (23/27)	NA
		3	87.5 (21/24)	87.5 (21/24)	NA	87.5 (21/24)	88.0 (22/25)	NA
		4	84.6 (22/26)	91.3 (42/46)	NA	88.0 (22/25)	88.5 (23/26)	NA
		5	81.5 (22/27)	60.9 (14/23)	NA	77.4 (24/31)	58.1 (18/31)	NA
	NPV, %	1	93.3 (42/45)	93.2 (41/44)	NA	93.2 (41/44)	93.3 (42/45)	NA
		2	93.5 (44/46)	93.3 (42/45)	NA	93.3 (42/45)	93.3 (42/45)	NA
		3	89.6 (43/48)	89.6 (43/48)	NA	89.6 (43/48)	91.5 (43/47)	NA
		4	91.3 (42/46)	89.8 (44/49)	NA	91.5 (43/47)	93.5 (44/46)	NA
		5	91.1 (41/45)	75.5 (37/49)	NA	95.1 (39/41)	80.5 (33/41)	NA
	Accuracy, %	1	90.3 (65/72)	88.9 (64/72)	n.s	88.9 (64/72)	90.3 (65/72)	n.s
		2	91.7 (67/72)	90.3 (65/72)	n.s	90.3 (65/72)	90.3 (65/72)	n.s
		3	88.9 (64/72)	88.9 (64/72)	n.s	88.9 (64/72)	90.3 (65/72)	n.s
		4	88.9 (64/72)	90.3 (65/72)	n.s	90.3 (65/72)	91.7 (67/72)	n.s
		5	87.5 (63/72)	70.8 (51/72)	0.01	87.5 (63/72)	70.8 (51/72)	0.01
	AUC	1	0.90	0.89	n.s	0.89	0.89	n.s
		2	0.92	0.91	n.s	0.91	0.91	n.s
		3	0.88	0.87	n.s	0.86	0.88	n.s
		4	0.88	0.89	n.s	0.89	0.91	n.s
		5	0.89	0.68	< 0.001	0.93	0.74	0.001

**Table 5:** Comparison of diagnostic performance in the PZ for clinically significant prostate cancer detection between DWI1000 and DWI2000 with short and long TR

Region	Parameter	Reader	DWI1000 with short TR	DWI1000 with long TR	*P* value	DWI2000 with short TR	DWI2000 with long TR	*P* value
PZ	Sensitivity, %	1	84.2 (16/19)	84.2 (16/19)	n.s	84.2 (16/19)	84.2 (16/19)	n.s
		2	84.2 (16/19)	84.2 (16/19)	n.s	84.2 (16/19)	84.2 (16/19)	n.s
		3	78.9 (15/19)	78.9 (15/19)	n.s	78.9 (15/19)	84.2 (16/19)	n.s
		4	78.9 (15/19)	78.9 (15/19)	n.s	78.9 (15/19)	84.2 (16/19)	n.s
		5	78.9 (15/19)	47.4 (9/19)	0.04	89.5 (17/19)	63.2 (12/19)	n.s
	Specificity, %	1	91.4 (32/35)	88.6 (31/35)	n.s	88.6 (31/35)	88.6 (31/35)	n.s
		2	91.4 (32/35)	88.6 (31/35)	n.s	88.6 (31/35)	88.6 (31/35)	n.s
		3	94.3 (33/35)	94.3 (33/35)	n.s	94.3 (33/35)	94.3 (33/35)	n.s
		4	88.6 (31/35)	94.3 (33/35)	n.s	91.4 (32/35)	91.4 (32/35)	n.s
		5	88.6 (31/35)	80.0 (28/35)	n.s	88.6 (31/35)	74.3 (26/35)	n.s
	PPV, %	1	84.2 (16/19)	80.0 (16/20)	NA	80.0 (16/20)	80.0 (16/20)	NA
		2	84.2 (16/19)	80.0 (16/20)	NA	80.0 (16/20)	80.0 (16/20)	NA
		3	88.2 (15/17)	88.2 (15/17)	NA	88.2 (15/17)	88.9 (16/18)	NA
		4	78.9 (15/19)	88.2 (15/17)	NA	83.3 (15/18)	84.2 (16/19)	NA
		5	78.9 (15/19)	56.3 (9/16)	NA	81.0 (17/21)	57.1 (12/21)	NA
	NPV, %	1	91.4 (32/35)	91.2 (31/34)	NA	91.2 (31/34)	91.2 (31/34)	NA
		2	91.4 (32/35)	91.2 (31/34)	NA	91.2 (31/34)	91.2 (31/34)	NA
		3	89.2 (33/37)	89.2 (33/37)	NA	89.2 (33/37)	91.7 (33/36)	NA
		4	88.6 (31/35)	89.2 (33/37)	NA	88.9 (32/36)	91.4 (32/35)	NA
		5	88.6 (31/35)	73.7 (28/38)	NA	93.9 (31/33)	78.8 (26/33)	NA
	Accuracy, %	1	88.9 (48/54)	87.0 (47/54)	n.s	87.0 (47/54)	87.0 (47/54)	n.s
		2	88.9 (48/54)	87.0 (47/54)	n.s	87.0 (47/54)	87.0 (47/54)	n.s
		3	88.9 (48/54)	88.9 (48/54)	n.s	88.9 (48/54)	90.7 (49/54)	n.s
		4	85.2 (46/54)	88.9 (48/54)	n.s	87.0 (47/54)	88.9 (48/54)	n.s
		5	85.2 (46/54)	68.5 (37/54)	n.s	88.9 (48/54)	70.4 (38/54)	n.s
	AUC, %	1	0.89	0.87	n.s	0.87	0.87	n.s
		2	0.89	0.88	n.s	0.87	0.88	n.s
		3	0.88	0.86	n.s	0.85	0.88	n.s
		4	0.84	0.85	n.s	0.85	0.88	n.s
		5	0.86	0.64	0.002	0.93	0.70	0.001

**Table 6:** Comparison of diagnostic performance in the TZ for clinically significant prostate cancer detection between DWI1000 and DWI2000 with short and long TR

Region	Parameter	Reader	DWI_1000_ with short TR	DWI_1000_ with long TR	*P* value	DWI_2000_ with short TR	DWI_2000_ with long TR	*P* value
TZ	Sensitivity, %	1	100 (7/7)	100 (7/7)	n.s	100 (7/7)	100 (7/7)	n.s
		2	100 (7/7)	100 (7/7)	n.s	100 (7/7)	100 (7/7)	n.s
		3	85.7 (6/7)	85.7 (6/7)	n.s	85.7 (6/7)	85.7 (6/7)	n.s
		4	100 (7/7)	85.7 (6/7)	n.s	100 (7/7)	100 (7/7)	n.s
		5	100 (7/7)	71.4 (5/7)	n.s	100 (7/7)	85.7 (6/7)	n.s
	Specificity, %	1	90.9 (10/11)	90.9 (10/11)	n.s	90.9 (10/11)	90.9 (10/11)	n.s
		2	100 (11/11)	100 (11/11)	n.s	100 (11/11)	100 (11/11)	n.s
		3	90.9 (10/11)	90.9 (10/11)	n.s	90.9 (10/11)	90.9 (10/11)	n.s
		4	100 (11/11)	100 (11/11)	n.s	100 (11/11)	100 (11/11)	n.s
		5	90.9 (10/11)	81.8 (9/11)	n.s	72.7 (8/11)	63.6 (7/11)	n.s
	PPV, %	1	87.5 (7/8)	87.5 (7/8)	NA	87.5 (7/8)	87.5 (7/8)	NA
		2	100 (7/7)	100 (7/7)	NA	100 (7/7)	100 (7/7)	NA
		3	85.7 (6/7)	85.7 (6/7)	NA	85.7 (6/7)	85.7 (6/7)	NA
		4	100 (7/7)	100 (6/6)	NA	100 (7/7)	100 (7/7)	NA
		5	87.5 (7/8)	71.4 (5/7)	NA	70.0 (7/10)	60.0 (6/10)	NA
	NPV, %	1	100 (10/10)	100 (10/10)	NA	100 (10/10)	100 (10/10)	NA
		2	100 (11/11)	100 (11/11)	NA	100 (11/11)	100 (11/11)	NA
		3	90.9 (10/11)	90.9 (10/11)	NA	90.9 (10/11)	90.9 (10/11)	NA
		4	100 (11/11)	91.7 (11/12)	NA	100 (11/11)	100 (11/11)	NA
		5	100 (10/10)	81.8 (9/11)	NA	100 (8/8)	87.5 (7/8)	NA
	Accuracy, %	1	94.4 (17/18)	94.4 (17/18)	n.s	94.4 (17/18)	94.4 (17/18)	n.s
		2	100 (18/18)	100 (18/18)	n.s	100 (18/18)	100 (18/18)	n.s
		3	88.9 (16/18)	88.9 (16/18)	n.s	88.9 (16/18)	88.9 (16/18)	n.s
		4	100 (18/18)	94.4 (17/18)	n.s	100 (18/18)	100 (18/18)	n.s
		5	94.4 (17/18)	77.8 (14/18)	n.s	83.3 (15/18)	72.2 (13/18)	n.s
	AUC	1	0.95	0.99	n.s	0.95	0.96	n.s
		2	1.00	1.00	n.s	1.00	1.00	n.s
		3	0.88	0.88	n.s	0.89	0.89	n.s
		4	1.00	1.00	n.s	1.00	1.00	n.s
		5	0.97	0.79	n.s	0.94	0.88	n.s

Data from patients who underwent radical total prostatectomy

*n.s.* not significant; *NA* not applicable; *PPV* positive predictive value; *NPV* negative predictive value; *AUC* area under the curve; *DWI* diffusion-weighted imaging; *TR* repetition time; *DWI*_*1000*_ DWI at a b-value of 1000 s/mm^2^; *DWI*_*2000*_ DWI at a b-value of 2000s/mm^2^

**Table 7:** Short TR DWI scores versus lesions with PI-RADS DWI score of 3 diagnosed with conventional long TR DWI

Criteria	DWI_1000_	DWI_2000_
Total cases with DWI score 3 in long TR	43/360 (11.9%)	26/360 (7.2%)
Short TR - upgrade to DWI score 4	12/43 (27.9%)	4/26 (15.4%)
Short TR - downgraded to DWI score 2	9/43 (20.9%)	7/26 (26.9%)
Short TR - same DWI score count	22/43 (51.2%)	15/26 (57.7%)
Short TR - accuracy (upgraded)	11/12 (91.7%)	5/7 (71.4%)
Short TR - accuracy (downgraded)	8/9 (88.9%)	2/4 (50%)

This analysis is based on the combined scores from five readers and pertains to data from patients who underwent radical total prostatectomy. The accuracy of upgrades and downgrades is displayed as the ratio of histologically confirmed diagnoses (PCa or benign lesions) to the number of cases with grade changes

*DWI* diffusion-weighted imaging; *PCa* prostate cancer; *TR* repetition time

## Discussion

In this study, we proposed a new approach called short TR DWI to improve diffusion contrast in PCa. To the best of our knowledge, this is the first study to incorporate features of PCa with shortened T1 relaxation times into DW images.

The SNR did not differ significantly between short and long TR acquisitions with a b-value of 1000 s/mm^2^. In contrast, CNR and visual score were significantly higher on DWI_1000_ with short TR. ADCs in PCa, NPT, and muscle showed strong correlations between short and long TR for b-values of 1000 and 2000s/mm^2^. No apparent bias was found for ADCs in PCa, NPT, and muscle between short and long TR for b-values of 1000 and 2000s/mm^2^ as limits of agreement for the mean difference contain zero. In a comparison of diagnostic performance by five readers using PI-RADS v2.1 DWI scores, four found the short TR DWI equivalent to the long TR DWI for both b1000 and b2000, while one reader observed a significantly higher diagnostic performance with the short TR DWI for both values. Furthermore, for lesions diagnosed with a PI-RADS DWI score of 3 using conventional long TR DWI, the short TR DWI upgraded or downgraded 48.8% of lesions in b1000 and 42.3% of lesions in b2000, thereby enhancing diagnostic accuracy. These results indicated that short TR DWI, especially in b1000, could exhibit improved clinical value. Two possible reasons for this can be: First, the changing magnetization transfer (MT) effect by increasing the number of packages (the number of packages is three in short TR and one in long TR). The slice-selective radiofrequency (RF) pulses for a specific slice act as off-resonance pulses in multi-slice sequences. Since the contribution of MT in multi-slice MRI increases with the number of off-resonance RF pulses applied during the TR interval, the effect could be more noticeable on DWI with long TR (the number of packages is one). Second, A shorter T1 value in PCa compared with NPT was found. As shown in Fig. 5, T1 saturation recovery graph has been indicated using T1 values of 1700 ms in PCa and 2100 ms in NPT, based on our results of mean T1 value. This could explain the larger signal difference between PCa and NPT in short TR and the higher signal in PCa because the shorter T1 value contributes to better diffusion contrast in short TR. Other studies utilizing this specific feature, the T1 value of PCa is lower than that of NPT, have reported that synthetic MRI with relaxometry measurements and MR fingerprinting could improve the diagnostic performance of PCa in PI-RADS category 3 lesions and TZ [10–13]. However, these methods require dedicated synthetic software and specific vendors and equipment. Although their reported methods may be superior in terms of diagnostic performance for PCa, short TR DWI had an advantage: it could be universally performed in most centers without requiring specific vendors, equipment, software or complex techniques, as it could be easily imaged by simply changing the TR settings.

Although a comparison of short and long TR acquisition with a b-value of 2000s/mm^2^ revealed that there was no significant difference in the visual score and CNR, short TR DWI tended to have better visual scores than did long TR DWI. Under high lesion contrast with an ultra-high b-value of 2000s/mm^2^, there was no significant difference in diffusion contrast between short and long TR. DWI with a b-value of at least 1400 s/mm^2^ is recommended in PI-RADS v2.1 [7]. However, the image quality of DWI with a b-value higher than 1400 s/mm^2^ might be compromised, depending on magnetic field strength and scanner performance. Therefore, it would be beneficial to acquire sufficient diffusion contrast using a standard b-value of 1000 s/mm^2^ with short TR instead of long TR. Moreover, calculated high b-value DW images in the prostate, obtained through extrapolation from acquired lower b-value data have been proposed [19, 20]. Therefore, improving diffusion contrast of low b-value using short TR would also be crucial for generating computed DWI with higher contrast in lesion.

Our study was limited by its retrospective, single-center design with a relatively small number of patients. The limited sample size in our study could potentially affect the generalizability and reliability of our results. Notably, in our evaluation of diagnostic performance, we focused on a relatively small cohort of nine patients who underwent total prostatectomy. We divided the PZ into six regions and the TZ into two separate regions. However, there's a possibility that the number of subdivisions for TZ was insufficient, potentially affecting the granularity of our results in that area. This constrained sample size may influence the power of our statistical tests and the accuracy of our effect size estimations. Studies with a small number of participants are more prone to the effects of random variation, leading to the possibility of overly optimistic findings or false positives. Furthermore, the visual score was assessed by a single reader using a 3-point scale. Thus, determining intra- and inter-reader reproducibility may be needed for more detailed visual scores such as a 5-point scale. These limitations may also have affected the generalizability of the results. Therefore, the current results should be validated in prospective multi-institutional clinical trials with multiple readers and a larger number of patients. In addition, the usefulness of short TR DWI with a b-value of 1000 s/mm^2^, in particular, should also be verified using a 1.5-T MRI scanner where it is difficult to ensure sufficient SNR for a high b-value DWI such as a b-value of 2000s/mm^2^. Given these limitations, our outcomes should be approached with caution, and a broader study is crucial to reaffirm our initial insights.

In conclusion, we have demonstrated a new approach for improving diffusion contrast in the prostate. The results indicated that short TR DWI_1000_ had better image quality than did conventional (long TR) DWI_1000_ in the prostate and may improve visualization of PCa for readers. Furthermore, the present results might have the potential for improving the value of computed DWI. In this study, the TR was shortened to enhance the signal intensity of PCa. To further improve the contrast of PCa, reducing the background signal was necessary, and one approach could be shortening the TE. In contrast to other organ cancers, PCa is known to have lower T2 values as well as T1 values compared with NPT. Therefore, future research is warranted for creating a new computed DWI optimized for detecting prostate cancer by varying not only TR but also TE, utilizing the specificity of T1 and T2 in the prostate.
